# Repurposing inhibitors of phosphoinositide 3-kinase as adjuvant therapeutics for bacterial infections

**DOI:** 10.3389/frabi.2023.1135485

**Published:** 2023-02-09

**Authors:** Renee Fleeman

**Affiliations:** Division of Immunity and Pathogenesis, Burnett School of Biomedical Sciences, College of Medicine, University of Central Florida, Orlando, FL, United States

**Keywords:** kinase inhibitor, drug resistant bacteria, adjuvant antibiotics, intracellular bacteria multiplication, bacterial invasion and survival

## Abstract

The rise in antimicrobial resistance and the decline in new antibiotics has created a great need for novel approaches to treat drug resistant bacterial infections. Increasing the burden of antimicrobial resistance, bacterial virulence factors allow for survival within the host, where they can evade host killing and antimicrobial therapy within their intracellular niches. Repurposing host directed therapeutics has great potential for adjuvants to allow for more effective bacterial killing by the host and antimicrobials. To this end, phosphoinositide 3-kinase inhibitors are FDA approved for cancer therapy, but also have potential to eliminate intracellular survival of pathogens. This review describes the PI3K pathway and its potential as an adjuvant target to treat bacterial infections more effectively.

## Introduction

We are approaching a future where the antibiotics that we rely on today will no longer be effective (Reardon, 2014). Consequently, there is an urgent need to find new therapeutics for multi drug resistant bacteria (De Oliveira et al., 2020). However, finding novel antibiotics to replace our existing arsenal has proven to be difficult (Silver, 2011). To answer this unmet need for novel antibacterials, there have been investigations into the potential of adjuvant therapeutics that inhibit bacterial drug resistance or improve immune system clearance of bacteria (Abdul-Ghani et al., 2006; Wright, 2016; Liu et al., 2019; Chang et al., 2021). These therapeutics do not directly kill the bacteria but allow for better clearance of the infection by the host and/or common antibiotics (Wright, 2016,) (Zumla et al., 2016; Chiang et al., 2018; Kaufmann et al., 2018), which could improve the therapeutic outcome in patients with severe or chronic bacterial infections (Kilinc et al., 2021; Wallis et al., 2022). Although not yet approved by the FDA, there are a variety of host directed therapeutics being investigated to help treat bacterial infections (Kaufmann et al., 2018; Barker et al., 2019; Liu et al., 2021; Wallis et al., 2022).

A host kinase that has promise as a potential adjuvant therapeutic target is phosphoinositide 3-kinase (PI3K) (Kiran et al., 2016; Paik et al., 2019; Adefemi et al., 2020; Kilinc et al., 2021). PI3K is dysfunctional in a wide range of cancers and inhibition of PI3K has proven effective to mitigate the carcinogenic upregulation of PI3K that leads to uncontrolled cellular growth (Yang et al., 2019). The advantage of repurposing PI3K inhibitors for infectious disease treatment is they are already FDA approved for cancer therapy and there is abundance research into PI3K inhibitors (Garber, 2014; Zhang et al., 2020; Mishra et al., 2021; Vanhaesebroeck et al., 2021; Richardson et al., 2022). There are several classes and isotypes of PI3Ks used ubiquitously throughout the body. However, class 1 and 3 PI3Ks are those involved specifically in macrophage killing of bacteria (Gillooly et al., 2001). In addition, class 1 PI3Ks are important for neutrophil migration, and it has been shown that aberrant migration in aged neutrophils is corrected in the presence of PI3K inhibitors (Sapey et al., 2014).

Inhibition of PI3K has potential as an adjuvant because bacterial pathogens manipulate the PI3K pathway to invade host cells and survive intracellularly (Krachler et al., 2011; Ogawa et al., 2011; Van Avondt et al., 2015; Ledvina et al., 2018). Depending on the stage of infection, bacterial manipulation of the PI3K pathway results in different outcomes that range from facilitating bacterial uptake into the host cells to inhibiting phagosome maturation and lysosomal fusion (Gillooly et al., 2001; Vergne et al., 2003; Vergne et al., 2004; Vergne et al., 2005; Cano et al., 2015). Obligate and non-obligate intracellular pathogens, *Chlamydia trachomatis* and *Mycobacteria tuberculosis* respectively, are examples of bacterial species that can survive and replicate intracellularly through manipulation of the PI3K pathway (Bai et al., 2014; Brooks et al., 2017; Sah and Lutter, 2020). PI3K manipulation by these species results in infections that are not only protected from host immune killing but are recalcitrant to antibiotic treatment (Hartkoorn et al., 2007; Bastidas and Valdivia, 2016; Ellis et al., 2019; Yu et al., 2020). In addition, facultative intracellular bacteria *Klebsiella pneumoniae* and *Salmonella typhimurium* can manipulate the PI3K pathway to avoid phagosome maturation and survive within macrophages for several days (Oelschlaeger and Tall, 1997; Cano et al., 2015; Bengoechea and Sa Pessoa, 2019). Utilizing PI3K inhibitors as adjuvants in combination with antibiotics for these infections would eliminate intracellular bacteria (Oghumu and Satoskar, 2013; Kimmey and Stallings, 2016) to allow more effective host bacterial clearance and antibiotic treatment (Cano et al., 2015). This review provides a brief overview of 1) PI3K function; 2) the various PI3K inhibitors; 3) how bacteria can manipulate PI3K to their advantage and 4) how PI3K inhibitors have potential as adjuvants to eliminate pathogens from their protective niches.

## Phosphoinositol 3-kinases

**Figure 1:**
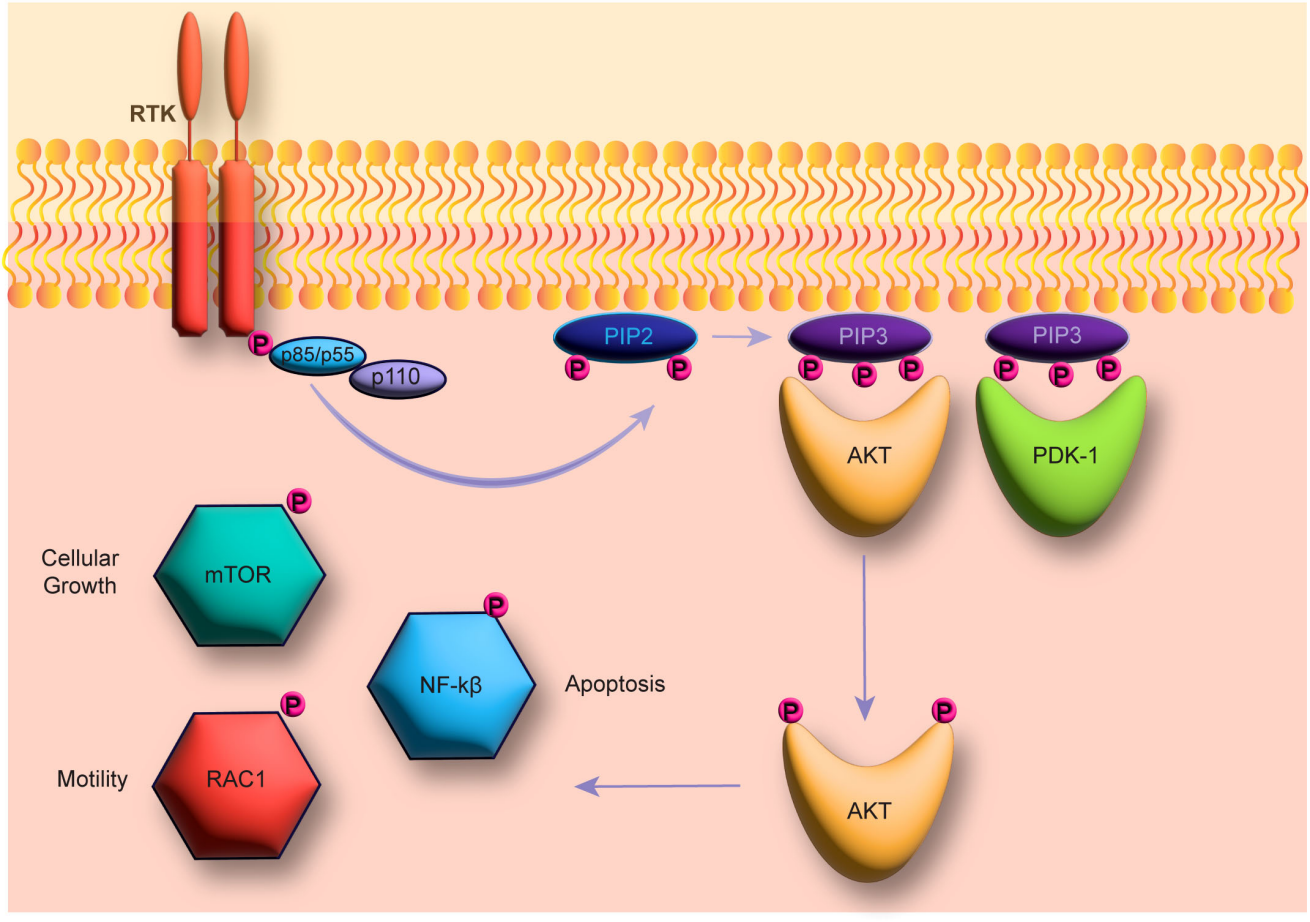
The PI3K pathway. The figure shows the PI3K pathway beginning with receptor tyrosine kinase (RTK) induction and phosphorylation of the catalytic PI3K subunit p110. The catalytic subunit then catalyzes the phosphorylation from PIP2 to PIP3, which in turn can then activate AKT and PDK-1 to activate downstream pathways (ie. mTOR, NF-kβ, and RAC-1).

Phosphoinositol 3-kinases (PI3Ks) are lipid kinases that reside in the plasma membrane of mammalian cells and consist of three subunits: two regulatory subunits p85 and p55; and a catalytic subunit p110 (Yang et al., 2019) (**Figure 1**). PI3Ks become activated after a transmembrane protein (ie. receptor tyrosine kinases (RTK)) signals the p85 regulatory subunit to bind and activate the p110 catalytic subunit (Yang et al., 2019). The catalytic subunit then phosphorylates phosphatidylinositol-4,5-bisphosphate (PtdIns-4,5-P_2_ or PIP2) to phosphatidylinositol-3,4,5-triphosphate (PtdIns-3,4,5-P_3_ or PIP3) (Fruman et al., 2017). Following phosphorylation, PIP3 is used as a secondary messenger to recruit and activate cytosolic proteins (ie. AKT and PDK-1) for a variety of purposes (Fruman et al., 2017) that vary between different cell types and PI3K isotypes (Leahy et al., 2012).

### PI3K isotypes and classes

There are 3 classes of PI3Ks and of these, class 1 is the most extensively studied due to having a major role in cancer development (Yang et al., 2019) (**Table 1**). This class is therefore the target of most therapeutic inhibitors in clinical development. Although not as well characterized as class 1, class 2 PI3Ks also have been shown to have role in cancer development (Gulluni et al., 2017; Gulluni et al., 2019; He et al., 2021). Specifically, this class has been shown to be important for migration of prostate cancer cells (Mavrommati et al., 2016). Class 3 PI3Ks are involved in membrane trafficking, endosome-lysosome maturation, and autophagosome formation (Jean and Kiger, 2014). Class 3 has been shown to play an important role in autophagy in the liver and heart (Jaber et al., 2012). However, this class has not yet been shown to have any role in disease or cancer (Jean and Kiger, 2014). Although PI3Ks are ubiquitous throughout the body, with select isotypes found in various compartment that have unique roles (Leahy et al., 2012).

**Table 1:** PI3K classes and isotypes.

Classes	Isotype	Location	Important role	Ref
Class I				
Class IA	p110**α**	ubiquitous	Cell signaling and growth	(Utermark et al., 2012)
	p110**β**	ubiquitous	Cell signaling and growth	(Utermark et al., 2012)
	p110**δ**	Immune cells	Cytokine-mediated B &T cell development and differentiation	(Hawkins and Stephens, 2015)
Class IB	p110**γ**	Immune cells	Myeloid chemotaxis, ROS/inflammatory secretion, and phagocytosis	(Nurnberg and Beer-Hammer, 2019)
Class II				
	PI3K-C2**α**	ubiquitous	Platelet membrane morphology, exocytosis, endocytosis and glucose transport	(Mazza and Maffucci, 2011; Valet et al., 2015; Wang et al., 2018)
	PI3K-C2**β**	ubiquitous	Clathrin-mediated pinocytosis	(Aung et al., 2019)
	PI3K-C2**γ**	Liver, pancreas, and reproductive organs	Dynamin-independent internalization	(Krag et al., 2010)
Class III				
	Vps34		Autophagy and endocytosis	(Juhasz et al., 2008; Jaber et al., 2012; Ohashi et al., 2020)

There are 4 class 1 PI3Ks isotypes that are named after their catalytic subunit proteins (**Table 1**). Class 1A consists of isotypes p110**α**, p110**β**, and p110**δ**, while class 1B consists of one isotype, p110**γ** (Yang et al., 2019). Isotypes p110**α** and p110**β** are constitutively expressed throughout the body and have many cellular functions (Leahy et al., 2012; Yang et al., 2019). Conversely, PI3K isotypes p110**δ** and p110**γ** are only found in immune cells and they can activate or repress downstream pathways (Fruman et al., 2017). Interestingly, p110**γ** are expressed in lymphocytes and are responsible for chemotaxis, making this specific PI3K isotype an excellent target for adjuvant immunotherapy to limit intracellular survival of pathogens (Leahy et al., 2012).

Class 2 PI3Ks have 3 isotypes: PI3K-C2**α**, PI3K-C2**β**, and PI3K-C2**γ** (Islam et al., 2020) (**Table 1**). Of these isotypes, PI3K-C2**α**, PI3K-C2**β** are found throughout the body, while PI3K-C2**γ** has been shown to be isolated to liver, pancreas, and reproductive organs (Islam et al., 2020). Isotype Class 2 PI3Ks are responsible for glucose uptake by the liver and have a role in blood pressure regulation (Koch et al., 2021). Studies with PI3K-C2**β** knock out mice revealed that because of its role in glucose uptake, this enzyme has potential as a target for diabetes treatment (Koch et al., 2021).

There are 4 class 1 PI3Ks isotypes that are named after their catalytic subunit proteins (**Table 1**). Class 1A consists of isotypes p110**α**, p110**β**, and p110**δ**, while class 1B consists of one isotype, p110**γ** (Yang et al., 2019). Isotypes p110**α** and p110**β** are constitutively expressed throughout the body and have many cellular functions (Leahy et al., 2012; Yang et al., 2019). Conversely, PI3K isotypes p110**δ** and p110**γ** are only found in immune cells and they can activate or repress downstream pathways (Fruman et al., 2017). Interestingly, p110**γ** are expressed in lymphocytes and are responsible for chemotaxis, making this specific PI3K isotype an excellent target for adjuvant immunotherapy to limit intracellular survival of pathogens (Leahy et al., 2012).

Class 2 PI3Ks have 3 isotypes: PI3K-C2**α**, PI3K-C2**β**, and PI3K-C2**γ** (Islam et al., 2020) (**Table 1**). Of these isotypes, PI3K-C2**α**, PI3K-C2**β** are found throughout the body, while PI3K-C2**γ** has been shown to be isolated to liver, pancreas, and reproductive organs (Islam et al., 2020). Isotype Class 2 PI3Ks are responsible for glucose uptake by the liver and have a role in blood pressure regulation (Koch et al., 2021). Studies with PI3K-C2**β** knock out mice revealed that because of its role in glucose uptake, this enzyme has potential as a target for diabetes treatment (Koch et al., 2021).

Class 3 has just a single isotype that is named after the catalytic subunit Vps34, with a corresponding regulatory subunit named Vsp15 (Jean and Kiger, 2014) (**Table 1**). Although insulin does not affect the activity of class 3 PI3Ks, it also has promise as a target for diabetes treatment because of its role in the feedback loop of glucose homeostasis (Nemazanyy et al., 2015). Interestingly, this therapy would target the regulatory subunit, not the usual therapeutic catalytic subunit because knocking down the regulatory subunit Vsp15, not the catalytic subunit Vsp34 has been shown to increase insulin sensitivity (Nemazanyy et al., 2015).

Considering the ubiquitous nature of PI3Ks throughout the body, when designing therapeutics for inhibition of PI3K classes, the specific isotype and its functions must be thoroughly investigated. For infectious disease adjuvants, focusing on classes and isotypes with functions in lymphocytes would result in a more specific therapeutic effect with less unwanted side effects. Therefore, designing specific inhibitors for class 1 isotypes p110**δ** and p110**γ** would be ideal for this purpose. Furthermore, it would be advantageous to repurpose PI3K inhibitors as adjuvants for bacterial infections with the extensive amount of research and development into inhibitors of class 1 PI3Ks as cancer therapeutics (Leahy et al., 2012).

### PI3K roles

PI3Ks are ubiquitous throughout the entire body and have been shown to be important for many physiological processes (Fruman et al., 2017). Research in the late 1980’s revealed that PtdIns-3,4,5-P3, the product of PI3K activation is central for malignant cancerous growth (Whitman et al., 1988; Auger et al., 1989). The PI3K/AKT/mTOR pathway is dysregulated in almost all cancer types leading to uncontrolled growth (Hoxhaj and Manning, 2020). Specifically, activation of the PI3K pathway in cancer cells is responsible for proliferation, invasion, metastasis, and angiogenesis (Rascio et al., 2021). Since the PI3K pathway is a driver of uncontrolled growth and spread of a variety of cancers, there are currently academic and clinical efforts in place to develop PI3K, AKT, and mTOR inhibitors as cancer therapeutics (Yang et al., 2019; Castel et al., 2021).

One important role for PI3K in the liver is regulation of glucose uptake and glycogen storage (Jean and Kiger, 2014; Koch et al., 2021). This makes PI3Ks possible targets for to treat the symptoms of diabetes (Maffei et al., 2018). Similarly, patients with a mutation in PI3K resulting in SHORT syndrome have difficulties with glucose homeostasis mimicking type 1 diabetes (Fruman et al., 2017). However, unlike diabetes, this defect does not affect the production of insulin but the ability of insulin to activate PI3K (Fruman et al., 2017). Its role in glucose regulation makes inhibiting PI3K for cancer therapy problematic because inhibition causes a release of glucose that in turn initiates the release of insulin. The consequence of this insulin release is re-activation of PI3K in tumor cells and ineffective chemotherapy (Fruman et al., 2017). However, only certain classes and isotypes have a role in glucose metabolism and their roles are unique (Maffei et al., 2018). For example, class 2 PI3Ks are activated by insulin (Koch et al., 2021), class 3 PI3Ks are involved in glucose feedback loop (Nemazanyy et al., 2015), and class 1A PI3Ks are the isotypes responsible for glucose regulation in the liver (Maffei et al., 2018). Furthermore, class 1A isotypes p110**α** and p110**β** are the major regulators of glucose in the liver, while class 1B p110**γ** regulates glucose in immune cells (Maffei et al., 2018).

Lastly, relevant to this review, PI3K has a major role in the innate and adaptive immune system. For professional phagocytes such as macrophages and neutrophils, PI3K plays a major role in both phagocytosis and killing of intracellular bacteria within phagosomes (Gillooly et al., 2001). Class 1A isotype p110**δ** and class 1B isotype p110**γ** and are the isotypes used specifically in immune cells (Okkenhaug, 2013). In addition to each isotype having a unique role, PI3Ks can activate or repress downstream genes depending on the cell type and associated receptor (Fruman et al., 2017). For example, class 1 Pi3Ks are involved in the phagosome cup formation, while class 3 PI3K is involved in the maturation (Koyasu, 2003). Specifically, class 1B isotype p110**γ** allows for neutrophil migration by producing PtdIns-3,4,5-P3 at the leading edge of the cell (Jean and Kiger, 2014). In addition, class 1A isotype p110**δ**, class 1B isotype p110**γ**, and class 3 PI3K Vps34 are responsible for bacterial clearance by immune cells (Thi and Reiner, 2012).

The extensive list of roles for PI3Ks make them attractive targets for cancer therapy, immune therapy, and diabetes treatment. When looking for therapeutic alternatives to complement our classical antibiotic therapy for drug resistant bacterial infections, targeting the PI3K specific isotypes is an intriguing possibility. Combination therapy with antibiotics and isotype selective PI3K inhibitors has the potential of increasing therapeutic outcome of drug resistant bacterial infections without interfering with PI3Ks in other cell types leading to unwanted side effects.

## PI3K inhibition

PI3K inhibitors are an exciting target for cancer therapy because upregulation of the PI3K/AKT/mTOR pathway is present in almost all cancers (Yang et al., 2019). PI3K upregulation is responsible for uncontrolled growth, increased chemotaxis, and invasiveness of cancer cells (Yang et al., 2019). Since the discovery of its important role in cancer development, PI3K inhibitors have been increasingly developed and optimized (Macara et al., 1984; Sugimoto et al., 1984; Whitman et al., 1985; Whitman et al., 1988).

### Broad-spectrum inhibitors

**Figure 2:**
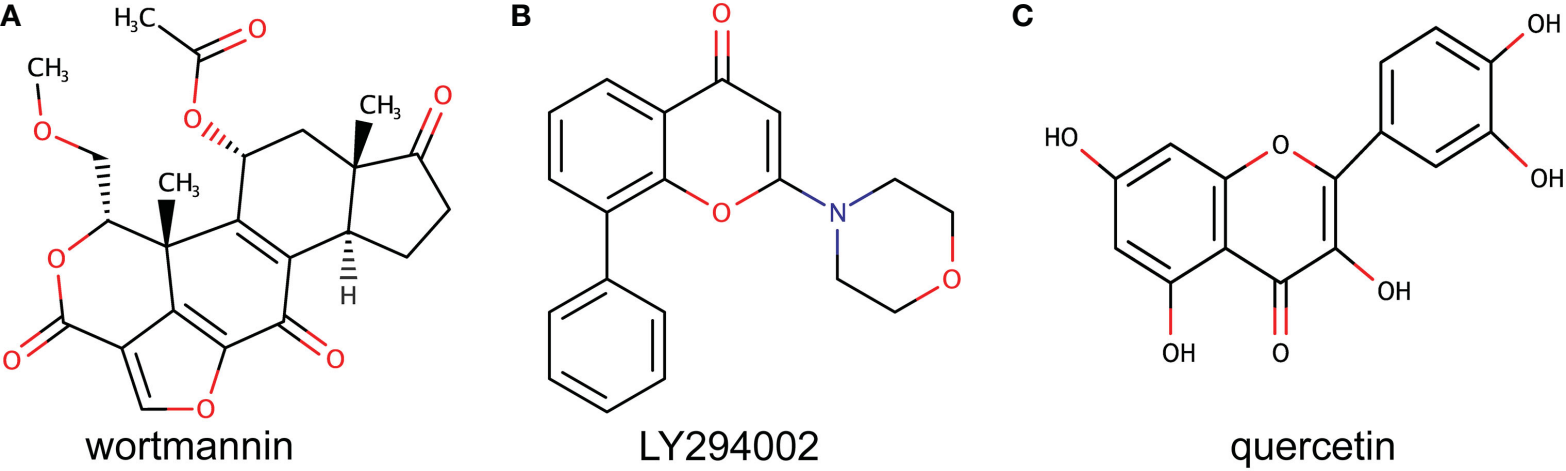
Original PI3K inhibitors. The figure shows the chemical structure of the first two discovered PI3K inhibitors. **(A)** shows wortmannin that was discovered from *Penicillium wortmannin.* **(B)** shows the chemical structure of the synthetic LY294002 designed based on the natural flavonoid quercetin shown in **(C)**.

In 1957 wortmannin, the first PI3K inhibitor was discovered after isolation from the fungal species *Penicillium wortmannin (* Cleary and Shapiro, 2010 *)* (**Figure 2A**). Similarly, Eli Lilly developed LY294002 as a reversible broad inhibitor of PI3k (Cleary and Shapiro, 2010) (**Figure 2B**). The development of LY294002 was based on optimizing the naturally occurring flavonoid quercetin (**Figure 2C**), that can inhibit a broad range of host kinases (Abdul-Ghani et al., 2006; Imai et al., 2012). These inhibitors have been used extensively in laboratories studying the cellular functions of PI3K but due to their poor solubility and physiochemical characteristics these inhibitors have not been used therapeutically (Cleary and Shapiro, 2010). After the discovery of PI3K inhibitors, efforts to improve their pharmacological characteristics resulted in several new inhibitors being developed (Cleary and Shapiro, 2010). Many studies have worked to improve the physiochemical characteristics of wortmannin and LY294002 through analog design resulting in more stable forms of wortmannin and more soluble analogs of LY294002 (Cleary and Shapiro, 2010).

In addition to the broad inhibitors of PI3K, therapeutics have been developed with the ability to bind both PI3K and the very similar catalytic subunit of mTOR (Cleary and Shapiro, 2010). Although they have not yet acquired FDA approval, there are dual PI3K and mTOR inhibitors in phase 3 of clinical development increasing the effectiveness of PI3K/AKT/mTOR pathway inhibition (Cleary and Shapiro, 2010; Yang et al., 2019). These have shown promise over therapeutics that solely inhibit mTOR, which has a negative feedback loop that activates PI3K and AKT when inhibited. However, it is uncertain at this point whether dual inhibition of both PI3K and mTOR is superior to the inhibition of PI3K alone (Cleary and Shapiro, 2010). In addition, multiple trials for dual inhibitors have been terminated due to low tolerability and adverse side effects (Yang et al., 2019). If the tolerability can be improved, perhaps dual PI3K/mTOR inhibitors could be beneficial for cancer therapy because studies have shown PI3K to be involved in chemotherapy resistance (Cleary and Shapiro, 2010; Garber, 2014).

### Isotype selective inhibitors

In recent years, isotype selective inhibitors have been more frequently pursued for development because they have been shown to have fewer side effects than broad-spectrum inhibitors (Cleary and Shapiro, 2010). For example, the PI3K isotype in the liver that is important for sending signals from the insulin receptor is p110**α**. Broad-spectrum PI3K inhibition can cause hyperglycemia by releasing extra glucose that in turn causes a large release of insulin (Fruman et al., 2017). Therefore, avoiding inhibition of PI3K isotype p110**α** can eliminate the hyperglycemic side effects that accompany broad PI3K inhibition (Fruman et al., 2017). In addition, it would be optimal to target specific isotypes when eliminating intracellular bacterial survival because p110**γ** and p110**δ** are the main PI3K isotypes for lymphocyte signaling (Cleary and Shapiro, 2010). This reveals great promise for avoiding unwanted side effects when using specific inhibitors to eliminate intracellular survival of bacterial pathogens.

Currently, there are isotype selective inhibitors that have been approved by the FDA (**Table 2**) and others are in stage 3 clinical trials (Garber, 2014). Specifically, in 2014 Gilead had the first PI3K isotype selective inhibitor Zydelig™ (Idelalisib) approved by the FDA for treatment of non-Hodgkin’s lymphoma, chronic lymphocytic leukemia, and follicular lymphoma (Garber, 2014). Zydelig™ is the only approved inhibitor specifically targeting the p110**δ** isotype of PI3K (Garber, 2014; Yang et al., 2019). To target PI3K p110**α**, Novartis acquired approval for Alpelisib™ (BYL719), for treatment of breast cancer (Lee et al., 2022). In addition to these very specific inhibitors, Copiktra^®^ (Duvelisib) and Aliqopa™ (Copanlisib) that have been approved shown to inhibit both p110**γ** and p110**δ** isotypes (Garber, 2014; Flinn et al., 2019; Yang et al., 2019). With these current approvals and more in various stages of clinical trials, there are many options to repurpose for adjuvant therapeutics.

**Table 2:** Approved PI3K inhibitors.

Name	Company	Isotype targeted	Commercial name	Ref
CAL-101/Idelalisib	Gilead	**δ**	Zydelig™	(Yang et al., 2015)
Bay80-6946/Copanlisib	Infinity	**γ** and **δ**	Aliqopa™	(Magagnoli et al., 2020)
IPI-145/Duvelisib	Gilead	**γ** and **δ**	Copiktra^®^	(Flinn et al., 2019)
BYL719/Alpelisib	Novartis	**α**	Piqray^®^	(Bogani et al., 2022)

Development of p110**γ** selective inhibitors has been slow because long term cancer treatment with these therapeutics can dampen the immune response to bacterial infections (Yang et al., 2019). However, acute inhibition of PI3K does not have the same effects on the immune system as long-term inhibition (Adefemi et al., 2020). Although T cell activation is inhibited, short term acute PI3K inhibition enhances the myeloid immune response to infections resulting in better infection control (Adefemi et al., 2020). Interestingly, it has been shown that chronic inflammation in elderly patients causes aberrant migration of neutrophils and PI3K inhibitors can help improve chemotaxis accuracy (Sapey et al., 2014). Aberrant neutrophil migration has also been observed in patients with severe sepsis (Patel et al., 2018). This exciting potential therapeutic application for PI3K inhibitors needs to be explored more thoroughly because the devastating mortality rates associated with sepsis (Vincent et al., 2002; Fleischmann-Struzek et al., 2020). Overall, selective inhibitors are optimal to repurpose as adjuvants for bacterial infection treatment because the PI3K isotypes used by the immune system are not ubiquitous throughout the body and the treatment length for bacterial infections is shorter than cancer therapy.

## Bacterial manipulation of PI3Ks

Bacterial pathogens can evade clearance by the host immune system by manipulating the cellular processes that facilitate clearance of invading organisms (Petit and Lebreton, 2022). There are a variety of mechanisms used by different bacterial species to survive, replicate, and hide within the host cells (Martinez et al., 2018). These virulence factors allow the pathogen to colonize and proliferate within a host organism and evade killing by antimicrobials (Liu et al., 2020; Petit and Lebreton, 2022). PI3Ks are targeted by many bacterial pathogens and therefore would be an advantageous target for host immune therapy and more effective treatment of chronic bacterial infections.

### Bacteria with traditional intracellular lifestyle

Intracellular bacteria can evade host immune clearance, reside, and replicate within macrophages and epithelial cells (Xue et al., 2010).. This ability to replicate and survive within the host is paramount to their success as a pathogen (Allwood et al., 2011; Mitchell et al., 2016). These pathogens are very difficult to treat because they can hide within the mammalian cells to evade antibiotic treatments (Kamaruzzaman et al., 2017; Tucker et al., 2021).

**Figure 3:**
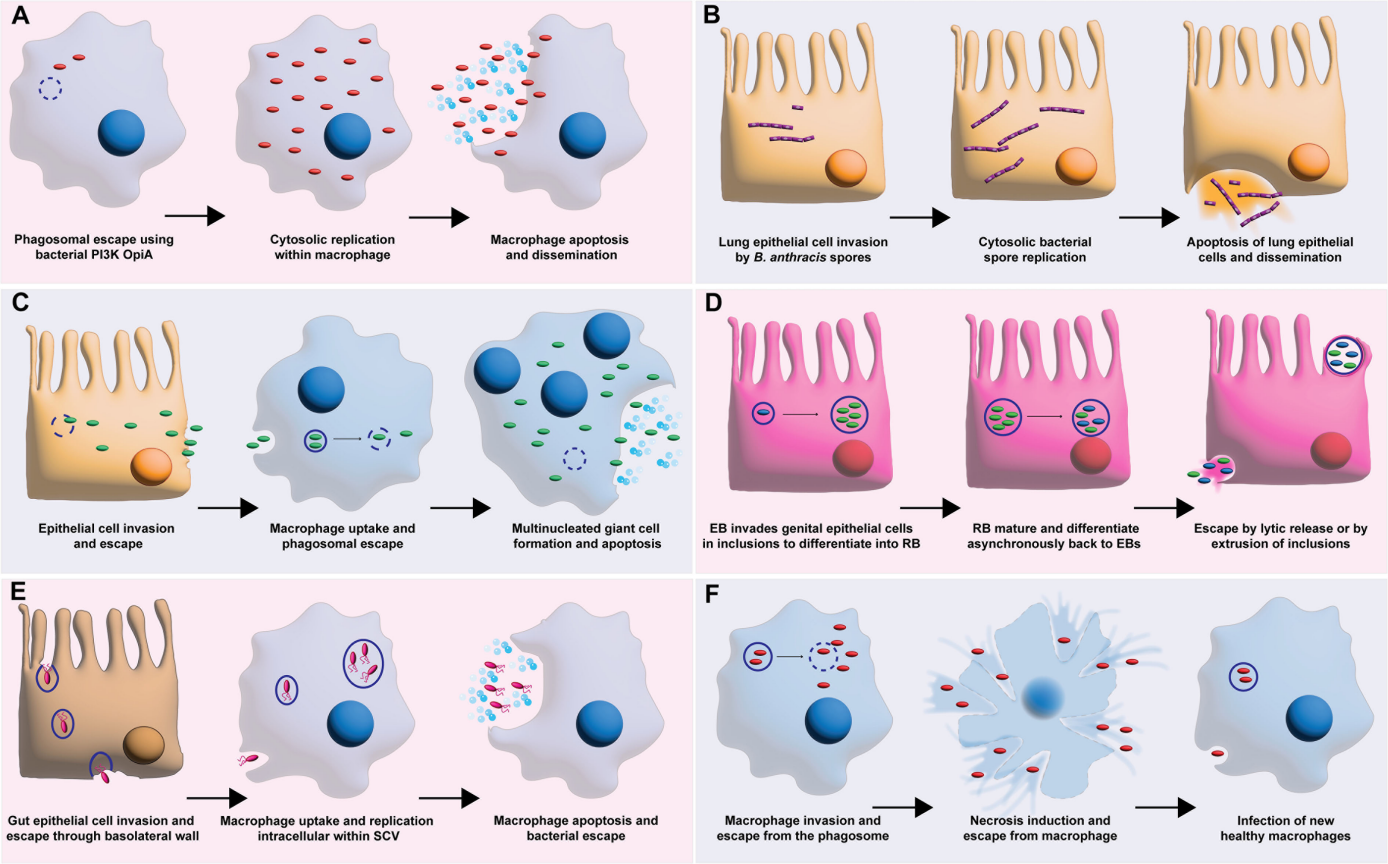
Bacterial intracellular survival strategies manipulating PI3K. **(A)** shows *F. tularensis* escape from the phagosome using its own bacterial PI3K OpiA. This allows for subsequent cytosolic replication, macrophage apoptosis, and bacterial dissemination. **(B)** shows *B. anthracis* spore invasion of lung epithelial cell attained by manipulation of PI3K. The resulting invasion leads to spore replication and epithelial cell apoptosis. **(C)** shows *B. pseudomallei* epithelial cell invasion with subsequent macrophage uptake facilitated by hijacking of PI3K pathway. Intracellular replication then leads to multinucleated giant macrophage cells, apoptosis, and bacterial escape. **(D)** shows *C. trachomatis* elementary body (EB) invasion of genital epithelial cells facilitated by manipulation of PI3K. This results in intracellular replication of reticulate bodies (RB) and infection spread. **(E)** shows *S. typhimurium* gut epithelial cell invasion facilitated by PI3K manipulation to invade macrophages to survive and replicate within the salmonella containing vacuole (SCV) before macrophage apoptosis leads to bacterial escape. **(F)** shows *M. tuberculosis* evasion of phagosome maturation leading to replication and escape from the phagosome, necrosis induction in the macrophage, and release to infect new macrophages.

*Francisella tularensis* is potential bioterrorism agent that causes fatal pneumoniae and no vaccine is available (Maurin, 2015). *F. tularensis* has an intracellular life cycle where it escapes from the phagosome and replicates within the macrophage cytosol (Celli and Zahrt, 2013; Ledvina et al., 2018) (**Figure 3A**). *F. tularensis* uses OpiA, its own bacterial PI3K to promote bacterial escape into the cytosol and this kinase has been shown to be resistant to the fungal PI3K inhibitor wortmannin (Ledvina et al., 2018). Furthermore, it has been suggested that additional intracellular bacteria, may harbor their own bacterial PI3K to facilitate phagosomal escape because OpiA family proteins (OFP) have been found in the genomes of intracellular replicating Legionella, Vibrio, and Rickettsia genera (Ledvina et al., 2018). Another potential bioterrorism agent, *Bacillus anthracis* replicates in the cytosol of host cells and manipulates the host PI3Ks to allow for intracellular replication (Xue et al., 2010). *B. anthracis* spores invade lung epithelial cells to replicate then escape to spread the infection (Xue et al., 2010) (**Figure 3B**). Xue et al. showed that treatment of A549 cells with the PI3K inhibitors LY294002 or wortmannin resulted in a drastic reduction in *B. anthracis* spore internalization, revealing the importance of PI3K activation by this species (Xue et al., 2010).

*Burkholderia pseudomallei* is a U. S. Tier 1 select agent for which there is no vaccine and high mortality rates are associated with the infections (Adefemi et al., 2020). This species is a facultative intracellular pathogen that can invade and replicate in both professional phagocytes and non-phagocytic cells (**Figure 3C**) (Adefemi et al., 2020). Specifically, *B. pseudomallei* can invade both epithelial cells and macrophages by using PI3K to hijack the host cell actin, ultimately creating multinucleated giant cells leading to apoptosis and escape (Hii et al., 2008; Pflughoeft et al., 2019). Interestingly, Ganesan et al. found that treatment with chloroquine in combination with doxycycline resulted in greater murine survival from *B. pseudomallei* infections by inhibiting an enzyme downstream to PI3K, glycogen synthase kinase-3β (Ganesan et al., 2020). This study shows the therapeutic benefit of repurposing host targeting drugs as adjuvants with a common antibiotic for treatment of bacterial infections (Ganesan et al., 2020).

The obligate intracellular Chlamydia species ability to manipulate host cell kinases is necessary for its success and survival as a pathogen (Sah and Lutter, 2020). More specifically, inhibition of PI3Ks by Chlamydia species allows for cell invasion, suppression of the host apoptosis and the acquisition of nutrients necessary for survival (Sah and Lutter, 2020) Once inside the host cell *Chlamydia trachomatis* differentiates from elementary bodies to reticulate bodies within inclusions that will eventually be either extruded from the cell or released by bacterial lysis after the cells mature and differentiate back into elementary bodies (**Figure 3D**) (Adefemi et al., 2020). In *C. trachomatis*, an effector protein TepP can recruit and activate PI3K on membranes to initiate bacterial invasion (Carpenter et al., 2017). Interestingly, the infectious elementary bodies of *C. trachomatis* that spread the infection have the highest amount of TepP proteins (Carpenter et al., 2017).

Salmonella species are facultative intracellular pathogens that have the ability to use multiple mechanisms to invade and replicate within host cells (Boumart et al., 2014) *Salmonella typhimurium* manipulates PI3K to pass through the gut epithelial lining, escape through the basolateral wall, and invade macrophages to replicate within the Salmonella containing vacuole (SCV) (**Figure 3E**) (Hurley et al., 2014). These species invade non-phagocytic cells by two mechanisms: the Zipper mechanism, shared by *Listeria monocytogenes* and the Trigger mechanism, shared by *Shigella flexneri (* Boumart et al., 2014 *)*. *S. typhimurium* manipulation of PI3K allows it to highjack macrophages, using them to disseminate the infection (Garcia-Gil et al., 2018). The *S. typhimurium* effector protein SopB activates PI3K pathway in B cells to facilitate survival (Roppenser et al., 2013; Garcia-Gil et al., 2018), by not allowing them to form the NLRCR4 inflammasome and fight the infection (Garcia-Gil et al., 2018).

*Mycobacterium tuberculosis* not only survives within macrophages but replicates within the phagosome (Liu et al., 2016). *M. tuberculosis* uses a recombinant leucine-responsive regulatory protein (rLpr) to increase activation of PI3K *via* the toll-like receptor 2 (TLR-2) (Liu et al., 2016). In addition, the marker for phagolysosome fusion Rab7 is targeted by *M. tuberculosis*, not allowing the phagosome to mature to the late stage (Nguyen and Yates, 2021). Following replication within the phagosome, the phagosomal membrane is permeabilized allowing the bacteria to escape to the cytosol, triggering necrosis of the macrophage and allowing escape of *M. tuberculosis* to infect other macrophages (**Figure 3F**) (Behar et al., 2010).

### Bacteria without traditional intracellular lifestyle

Some pathogens are not all traditionally considered when investigating bacterial survival within host cells (Sendi and Proctor, 2009; Silva, 2012). However, these pathogens can manipulate the immune system to their advantage to allow survival and spread of the infection (Silva, 2012; Silva and Pestana, 2013). Therefore, when considering adjuvant PI3K therapy these pathogens should also be considered potential targets.

**Figure 4:**
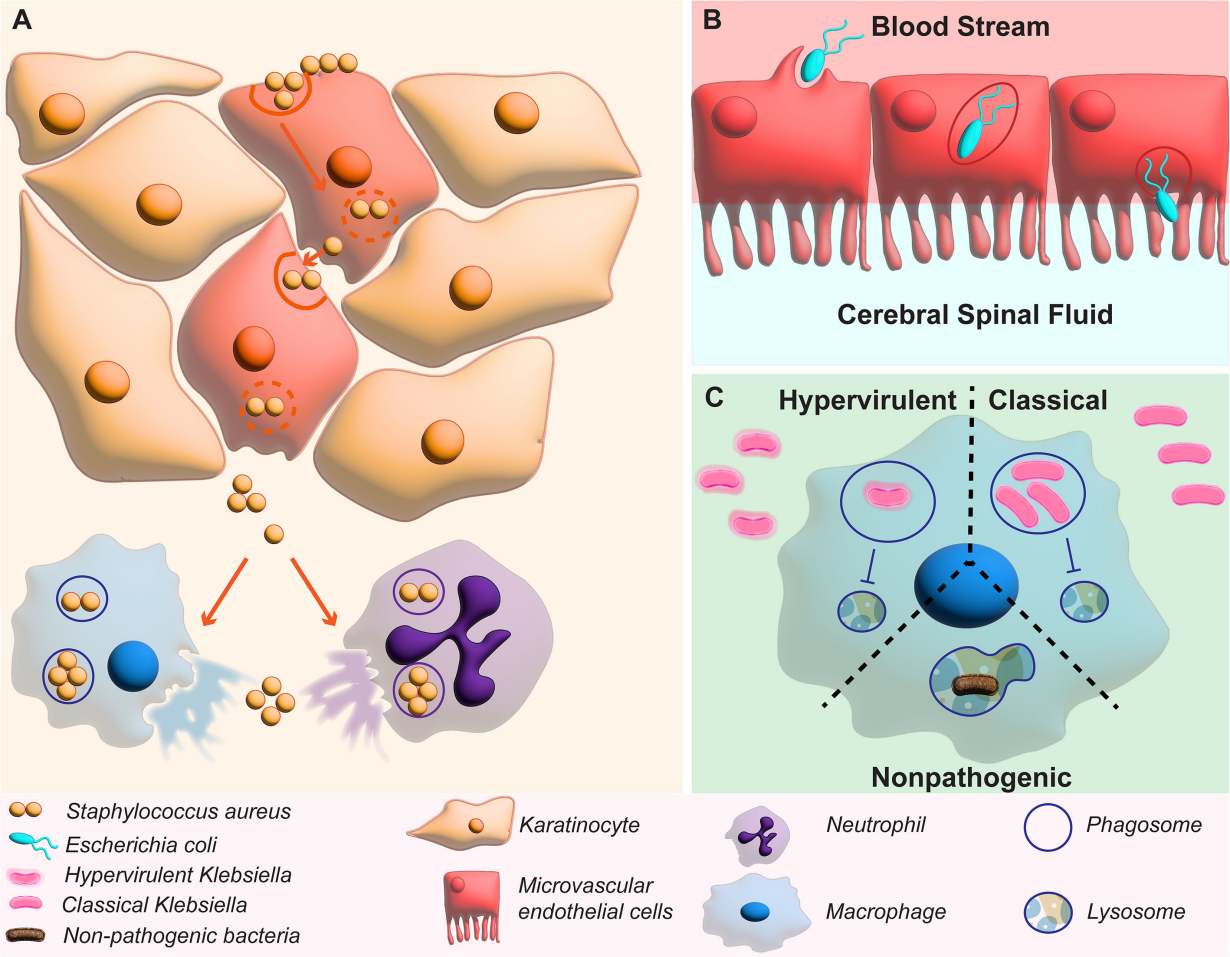
Manipulation of PI3K by non-traditional intracellular bacteria. **(A)** shows how manipulation of PI3K by *S. aureus* leads to invasion of keratinocytes of the dermis to spread the infection and can replicate within phagocytes in macrophages and neutrophils. **(B)** shows *E. coli* invasion of microvascular endothelial cells to pass the blood brain barrier. **(C)** shows how activation of PI3K by hypervirulent and classical *K. pneumoniae* inhibit phagolysosome fusion that usually kills non-pathogenic bacteria.

*Staphylococcus aureus* is a dangerous pathogen with community acquired infections easily spread due to their ability to invade healthy individuals (Boyle-Vavra and Daum, 2007; DeLeo et al., 2009; Bukharie, 2010; Yoong and Pier, 2010; Turner et al., 2019). Skin infections caused by *S. aureus* can quickly invade and disseminate leading to systemic infection with sepsis (Edwards et al., 2010). Although traditionally thought of as an extracellular pathogen, *S. aureus* has been shown to invade non-phagocytic mammalian cells (Nakagawa et al., 2017). For example, keratinocyte invasion by *S. aureus* induces lysis continued invasion of the dermis layer. When engulfed by macrophages and neutrophils partial evasion of phagolysosome killing leads to escape and dissemination of infection (Oviedo-Boyso et al., 2011; Hommes and Surewaard, 2022) (**Figure 4A**). Interestingly, *S. aureus* internalization decreases in bovine epithelial cells when treated with PI3K inhibitors (Oviedo-Boyso et al., 2011). *Escherichia coli* K1 has been shown to invade endothelial cells to cross the blood brain barrier and cause bacterial meningitis (Reddy et al., 2000). *E. coli* has been shown to manipulate the human brain microvascular endothelial cells and force micropinocytosis (Loh et al., 2017) (**Figure 4B**). Attachment to the epithelial cell lining of the blood brain barrier by *E. coli* activates several signaling pathways including the PI3K pathway (Loh et al., 2017).

A close relative to *E. coli*, *K. pneumoniae* is historically know an extracellular pathogen, unable to survive for extended periods in intracellular compartments like its close relative *E. coli* (Belon and Blanc-Potard, 2016). However, studies have now shown that *K. pneumoniae* is able to survive for up to 48 hours within the vacuole once engulfed by macrophages (Oelschlaeger and Tall, 1997). This vacuole, named the *Klebsiella* containing vacuole (KCV) does not fuse with lysosomes and therefore deviates from the canonical endocytic pathway used by macrophages to clear engulfed pathogens (Bengoechea and Sa Pessoa, 2019) (**Figure 4C**). This lack of maturation of the phagosome also results in less activation of the adaptive immune system because less antigens are presented on the surface of macrophages (Cano et al., 2015). Although it is unclear the factor that allows *K. pneumoniae* to persist within the phagosome, the capsule does not appear to play a major role because both hypervirulent and classical *K. pneumoniae* can manipulate the PI3K pathway to stop phagosome maturation by inhibiting fusion with the lysosome compartment (**Figure 4C**) (Cano et al., 2015). These classical intracellular pathogens have been shown to display less intracellular survival in the presence of AKT inhibitors targeting the enzyme immediately downstream to PI3K in the PI3K/AKT/Rab14 axis (Bengoechea and Sa Pessoa, 2019). In addition, it has recently been revealed that *K. pneumoniae* manipulates PI3K through the mammalian protein SARM1 (sterile **α** and HEAT armadillo motif-containing protein) revealing a potential target upstream for future therapeutic development (Feriotti et al., 2022). With the many bacterial species that manipulate PI3K to invade the host and survive intracellularly, repurposing PI3K inhibitors to release them from their protective niches would be beneficial to allow the host immune system and antibiotics to be more effective at treating these infections.

## PI3K inhibitors as adjuvants for bacterial infections

With the variety of pathogens that manipulate the PI3K pathway to evade host immune killing (Krachler et al., 2011; Cano et al., 2015; Liu et al., 2016; Lacoma et al., 2017; Garcia-Gil et al., 2018), investigating the potential as adjuvant therapeutics to eliminate intracellular bacterial survival is important for the fight against drug resistant bacterial species. PI3K inhibitors provided in a short, acute dose can promote bacterial pathogen clearance (Adefemi et al., 2020). Using PI3K inhibitors can release the pathogens from their niches used to hide from the immune system (Wong et al., 2019) and disseminate infection (Chen et al., 2002). In combination with antibiotics, PI3K inhibitors have the potential to behave as adjuvants allowing more effective antibiotic therapy at lower doses.

### Phagolysosome fusion inhibition

**Figure 5:**
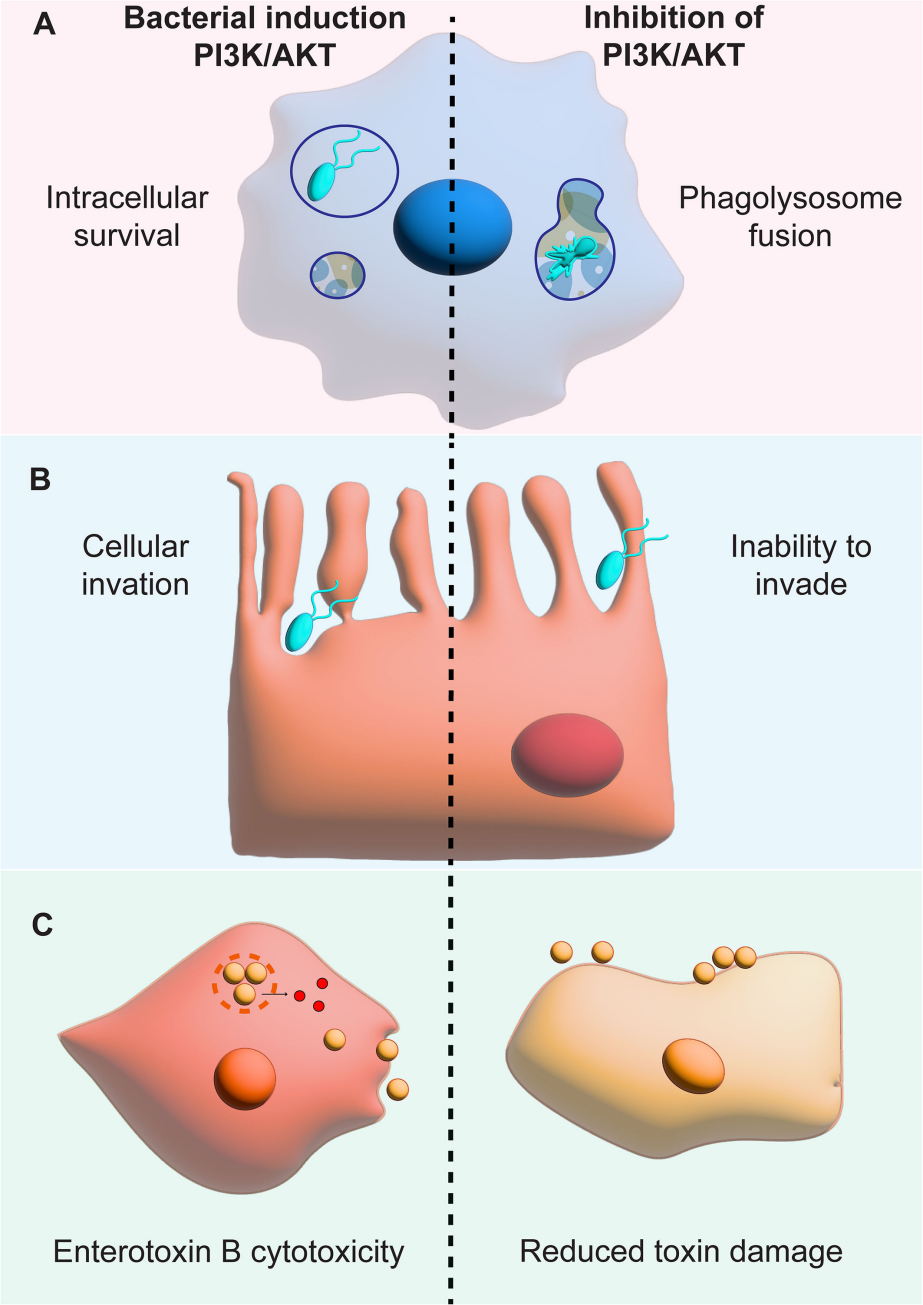
PI3K inhibition decreases bacterial invasion and survival within host cells. The figure shows the effects of PI3K manipulation next to the effects of PI3K inhibition. **(A)** shows that bacterial PI3K manipulation used by *S. typhimurium, M. tuberculosis and K. pneumoniae* to inhibit phagolysosome fusion can be stopped by PI3K inhibitors leading to fusion and bacterial death. **(B)** shows how the invasion of epithelial cells by *E. coli* is stopped by PI3K inhibitors. **(C)** reveals the benefits of PI3K inhibition on the keratinocyte survival in the presence of the Enterotoxin B toxin.

Studies have shown that acute PI3K treatment can improve the early-stage progression of infections (Adefemi et al., 2020). Many genera of bacteria manipulate PI3K to avoid phagolysosome fusion and PI3K inhibitors allow for efficient fusion of the lysosome and bacterial clearance (**Figure 5A**). This has been used to show that PI3K/AKT inhibition can eliminate intracellular *S. typhimurium* and *M. tuberculosis* (Bengoechea and Sa Pessoa, 2019). Interestingly, inhibition of PI3K in *M. tuberculosis* by isotype specific inhibitors has the potential to decrease the characteristic late-stage infection IL-17A induced pathology by interfering with Th17 differentiation (Leisching, 2019). Shapira et al. performed high-content screening of kinase inhibitors and found inhibition of PI3K controls autophagy and apoptosis decreasing intracellular survival (Shapira et al., 2020). This effect has also been seen with the facultative intracellular *K. pneumoniae* that manipulates the PI3K pathway to avoid phagolysosome fusion of late-stage endosomes (Cano et al., 2015). Treatment with a PI3K inhibitor revealed a decrease in *K. pneumoniae* intracellular survival within macrophages (Cano et al., 2015; Bengoechea and Sa Pessoa, 2019). Inhibiting bacterial pathogen survival in host immune cells has great potential for treatment of chronic infections.

### Decreasing invasion and survival in non-phagocytic cells

PI3K inhibition also has the potential to decrease bacterial invasion and survival within non-phagocytic cells like epithelial cells. Testing PI3K inhibitors when infecting intestinal cells with *S. typhimurium*, Huang et al. revealed that this species uses PI3K activation to decrease inflammation. This decreased inflammation allows *S. typhimurium* to survive and PI3K inhibition led to decreased bacterial survival within intestinal epithelial cells (Huang et al., 2005). Like *S. typhimurium*, PI3K manipulation is also important for epithelial cell invasion by *Helicobacter pylori* and *Listeria monocytogenes* revealing the potential of PI3K inhibitors for a variety of intracellular pathogens (**Figure 5B**) (Booth et al., 2003). Furthermore, the penetration of the blood brain barrier by *E. coli* leading to meningitis can be stopped by using PI3K inhibitors (Loh et al., 2017). These studies reveal the potential of PI3K therapeutics for non-phagocytic cells revealing a broader application for adjuvants for a variety of infections and bacterial species.

### Rescuing from toxic effects of bacterial infections

PI3K inhibitors have also shown promise in treating pathogens that do not have a true intracellular stage (Whitman et al., 1988; Cano et al., 2015; Yang et al., 2019). With these infections, inflammation and toxic damage can be mitigated by using PI3K inhibitors. For example, the natural PI3K inhibitor deguelin reduces Staphylococcal Enterotoxin B induction of T-cell proliferation toxicity (Whitfeild SJ et al., 2017) (**Figure 5C**). However, more work is needed to understand all the potential effects of PI3K treatment for bacterial infections. For example, the treatment of an *E. coli* induced endotoxemic mice with PI3K inhibitors lead to a decrease in mouse survival because of increased LPS-induced inflammation (Schabbauer et al., 2004). This data reveal that PI3K inhibitors can also be repurposed to protect from the effects of bacterial toxins during infection but much research is needed to pursue these applications.

## Conclusions

This review provides an overview of the role of PI3K pathway in intracellular bacterial survival and how repurposing PI3K inhibitors can potentially help eliminate these difficult to treat bacterial infections. PI3K inhibition in combination with antibiotics and the host immune system can lead to more effective treatment of many bacterial infections. However, much research is needed to explore the potential of PI3K inhibitors as adjuvant therapeutics for intracellular bacterial pathogens.

## Author contributions

The review design, writing, figures, and editing was performed by RF.
